# Dual population-level processes contribute to polyclonal ceftiofur heteroresistance in swine-derived *Escherichia coli*

**DOI:** 10.1080/21505594.2026.2711528

**Published:** 2026-07-30

**Authors:** Junling Cui, Zhongyi Fang, Qiuru Chen, Kun He, Shaochuan Xing, Zhengyu Wei, Dandan He, Gongzheng Hu, Xiaoyuan Ma, Li Yuan

**Affiliations:** a College of Veterinary Medicine, Henan Agricultural University, Zhengzhou, PR China; b Henan Institute of Technical Research on Agricultural Product, Zhengzhou, PR China; c Key Laboratory of Quality and Safety Control of Poultry Products (Zhengzhou), Ministry of Agriculture and Rural Affairs, Zhengzhou, PR China; d Ministry of Education Key Laboratory for Animal Pathogens and Biosafety, Zhengzhou, PR China

**Keywords:** Ceftiofur, polyclonal heteroresistance, *bla*_CTX-M_, population dynamics

## Abstract

Antimicrobial resistance represents a major global health challenge. In veterinary medicine, ceftiofur is widely used to treat bacterial infections, yet its efficacy has been increasingly compromised by the dissemination of extended-spectrum *β*-lactamase (ESBL) genes such as *bla*_CTX-M_. Although heteroresistance has been widely reported, its role in ceftiofur resistance, particularly in swine-derived *Escherichia coli*, remains poorly understood. Here, we identified four polyclonal ceftiofur heteroresistance (PCHR) *E. coli* isolates from swine, each comprising genetically distinct resistant and susceptible subpopulations. Whole-genome sequencing showed that all resistant subpopulations carried *bla*_CTX-M_ genes, and conjugation assays demonstrated that *bla*_CTX-M_-carrying plasmids were transferable. Notably, in the resistant subpopulation EP91A, a chromosomal *bla*_CTX-M_-containing fragment was identified in the transconjugant plasmid pTEP91A-1, with sequence features consistent with a possible *IS1380*-associated recombination event. Under ceftiofur pressure, resistant subpopulations expanded in all four PCHR isolates, although the associated population-level processes differed among isolates, including plasmid-mediated transfer and differential expansion of preexisting resistant subpopulations. Under ceftiofur-free conditions, resistant subpopulations were maintained at different levels, with EP70A and EP91A reaching higher proportions and stabilizing at approximately 43%, suggesting clone-associated population dynamics under antibiotic-free conditions. Transcriptomic analysis further identified clone-associated transcriptional differences between EP91A and EP91B across pathways related to environmental sensing, metabolism, transport, and cellular processes, providing hypothesis-generating observations. Collectively, these findings suggest that PCHR in these selected swine-derived *E. coli* isolates is associated with genetic heterogeneity, transferable *bla*_CTX-M_-carrying plasmids, and distinct population dynamics, providing insights into ceftiofur resistance expansion and maintenance in heterogeneous bacterial populations.

## Graphical abstract

**Figure:**
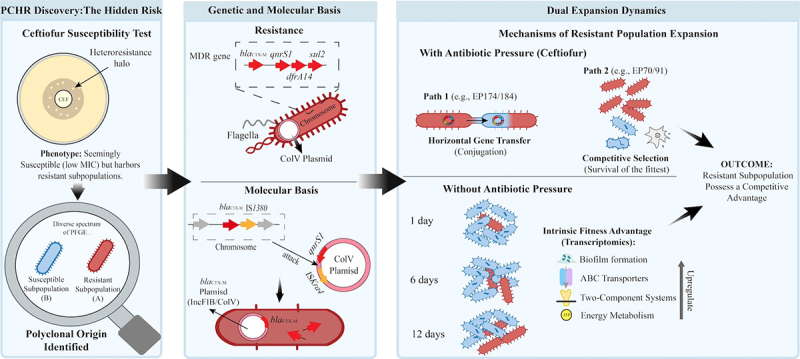

## Introduction

Ceftiofur (CEF), a third-generation cephalosporin developed exclusively for veterinary use, remains an important therapeutic agent for bacterial infections in food-producing animals, particularly respiratory and soft tissue diseases in swine. However, its extensive use has been accompanied by the increasing emergence of resistance. The widespread dissemination of *bla*_CTX-M_-type extended-spectrum *β*-lactamase (ESBL) genes exemplifies this concern. A study conducted in China from 2012 to 2020 reported that more than 60% of 358 avian *E. coli* isolates from deceased chickens harbored *bla*_CTX-M_, with all positive strains exhibiting resistance to third-generation cephalosporins [1]. In addition, Mwakyoma et al. demonstrated widespread horizontal transfer of *bla*_CTX-M_ among *Enterobacteriaceae* from human, animal, and environmental sources, highlighting the risk of cross-sector dissemination [2]. While the spread of *bla*_CTX-M_-producing *E. coli* is a well-established contributor to ceftiofur treatment failure [3], increasing attention has been directed toward additional resistance phenotypes that may complicate antimicrobial susceptibility testing and treatment outcomes, including heteroresistance.

Heteroresistance refers to a phenomenon in which a seemingly homogeneous bacterial population contains a minor subpopulation with significantly higher resistance to an antibiotic than the dominant susceptible population. This hidden diversity poses a major challenge, as standard susceptibility testing often classifies such populations as susceptible, while failing to detect resistant subpopulations [4–7]. Based on genetic relatedness, heteroresistance can be broadly categorized into monoclonal heteroresistance (MHR), arising from genetic instability or phenotypic variation within a single clone, and polyclonal heteroresistance (PHR), resulting from the coexistence of multiple genetically distinct clones within a single isolate culture or bacterial population [8]. Clinically, heteroresistance is concerning because preexisting resistant subpopulations can be selectively enriched during antibiotic exposure, potentially contributing to treatment failure and the emergence of stable high-level resistance [9–12]. Consistent with this concern, heteroresistance has been reported in a wide range of major pathogens, including *Escherichia coli, Staphylococcus aureus*, *Acinetobacter baumannii*, *Pseudomonas aeruginosa*, and *Klebsiella pneumoniae*, and across multiple *β*-lactam antibiotics ranging from cefepime to carbapenems [13–20]. Despite this growing body of evidence, heteroresistance remains poorly characterized for veterinary-exclusive antimicrobials such as ceftiofur.

In our preliminary investigation of swine-derived *E. coli*, we observed intra-isolate variation in ceftiofur MICs, with differences of up to 2048-fold, a feature consistent with ceftiofur heteroresistance (CHR). Pulsed-field gel electrophoresis (PFGE) analysis further suggested that several isolates (EP70, EP91, EP174, and EP184) may represent cases of polyclonal ceftiofur heteroresistance (PCHR). However, although heteroresistance in *E. coli* has been documented across multiple classes of antibiotics, CHR in swine-derived *E. coli* remains poorly understood. In particular, the genetic features of resistant subpopulations, their potential for resistance gene transfer, and the population-level dynamics of resistant and susceptible subpopulations under antibiotic and antibiotic-free conditions remain unclear.

Therefore, this study was designed to investigate PCHR in selected swine-derived *E. coli* isolates. Specifically, we aimed to: (i) characterize the polyclonal nature of these isolates using phenotypic assays, PFGE, and whole-genome sequencing (WGS); (ii) examine resistance gene profiles and plasmid-associated features, with a focus on *bla*_CTX-M_-carrying elements and their transferability; and (iii) evaluate population dynamics of resistant subpopulations under ceftiofur exposure and antibiotic-free conditions. This work provides a case-series analysis of PCHR in swine-derived *E. coli* and may offer insights into resistance expansion and maintenance within heterogeneous bacterial populations under the tested conditions.

## Materials and methods

### Bacterial strains

A total of 453 non-repetitive *E. coli* isolates were collected from multiple pig farms in Henan province, China, between 2018 and 2024. During initial isolation, presumptive *E. coli* colonies were individually picked from primary culture plates and purified by single-colony streaking before species identification. All isolates were identified using matrix-assisted laser desorption/ionization time-of-flight mass spectrometry (MALDI-TOF MS). For each isolate, one purified and identified colony was inoculated into fresh Luria-Bertani (LB) broth and cultured at 37°C with shaking to an OD_600_ of 0.5, after which multiple aliquots were prepared and stored at −80°C as purified culture stocks for subsequent disk diffusion, population analysis profile (PAP), *in vitro* time-kill assay, and population dynamics, and other downstream assays. *E. coli* ATCC 25,922 was used as the quality control strain, and a rifampin-resistant derivative of *E. coli* C600 was used as the recipient strain in conjugation assays.

### Preliminary screening of heteroresistant E. coli

The susceptibility of all 453 purified *E. coli* isolates to ceftiofur was determined using the standard disk diffusion method [21]. Commercial ceftiofur disks (30 μg; Liofilchem S.r.l., Roseto degli Abruzzi, Italy) were used. Briefly, preserved purified cultures were recovered and incubated overnight at 37°C before testing. The inhibition zone diameters were measured and interpreted according to the CLSI breakpoint for ceftiofur against *E. coli*, with an inhibition zone diameter of ≤17 mm defined as resistant [22,23]. Isolates exhibiting scattered colonies or a halo-like growth pattern within the inhibition zone were considered suspected CHR isolates. Each assay was independently repeated at least three times.

### Population analysis profile assays

PAP assays were performed on the four suspected CHR *E. coli* isolates EP70, EP91, EP174, and EP184, using preserved purified cultures derived from the initial single-colony purification, according to a previously described method with modifications [7]. Briefly, overnight cultures were diluted in fresh LB broth (Beijing Aoboxing Bio-Tech Co., Ltd., China) and incubated at 37°C with shaking until reaching an OD_600_ of 0.7. Subsequently, the cultures were serially diluted in saline, and 100 μL aliquots were spread onto Mueller-Hinton agar (MHA; Beijing Aoboxing Bio-Tech Co., Ltd., China) plates containing ceftiofur at concentrations of 0, 1, 2, and 4 μg/mL, corresponding to 0, 4×, 8×, and 16× the parental MIC of each suspected CHR isolate, respectively. After 48 h of incubation at 37°C, the colonies were counted. The bacterial growth frequency at each antibiotic concentration was calculated relative to the colony count on the drug-free plate. An isolate was confirmed as CHR if it grew on plates containing ceftiofur at ≥4× MIC at a frequency of ≥1 × 10^−7^ [21]. *E. coli* ATCC 25,922 was used as the control strain. Experiments were performed in three independent assays.

### In vitro time-kill assay

Following confirmation by PAP assays, *in vitro* time-kill assay was performed on the four CHR *E. coli* isolates according to established methods [24,25]. Preserved purified cultures were diluted 1:100 in preheated Mueller-Hinton broth (MHB, Beijing Aoboxing Bio-Tech Co., Ltd., China) containing ceftiofur at 4× MIC and incubated at 37°C. Over a 48 h period, samples were periodically collected, serially diluted in 0.9% saline, and plated onto antibiotic-free MacConkey agar (Beijing Aoboxing Bio-Tech Co., Ltd., China). After 18 h of incubation at 37°C, colony-forming units (CFU) were counted, and viable bacterial counts were expressed as log_10_ CFU/mL. The experiment included three independent biological replicates.

### Pulsed-field gel electrophoresis (PFGE)

The clonal relationships between the ceftiofur-resistant subpopulations (EP70A, EP91A, EP174A, EP184A) and their corresponding susceptible counterparts (EP70B, EP91B, EP174B, EP184B) were analyzed by PFGE according to an established protocol [26]. Specifically, total DNA was digested with the *XbaI* restriction enzyme (TaKaRa Bio Inc., Shiga, Japan), and embedded in low-melting-point agarose plugs (Bio-Rad Laboratories, Hercules, CA, USA). PFGE was then performed using a CHEF-MAPPER system (Bio-Rad Laboratories, Hercules, CA, USA), with *Salmonella* serotype Braenderup H9812 strain used as a molecular size marker. Banding patterns were visualized on the gel imaging system (Bio-Rad Laboratories, Hercules, CA, USA) and analyzed with BioNumerics v6.6 (Applied Math NV, Sint-Martens-Latem, Belgium).

### Next-generation sequencing and bioinformatic analysis

Genomic DNA from the resistant and susceptible subpopulation pairs (EP70A/B, EP91A/B, EP174A/B, EP184A/B) was sequenced on an Illumina NextSeq 500 platform (Illumina, Inc., San Diego, CA, USA). Raw reads were subjected to quality control using FastQC (v0.11.9) and adapter trimming using TrimGalore (v0.6.6). High-quality reads were de novo assembled into contigs using MEGAHIT (v1.2.9) [27]. Subsequent bioinformatic analyses included open-reading frame (ORF) prediction with Prodigal (v2.6.3), serotype identification using SeroTypeFinder (v2.0) [28], multilocus sequence typing (MLST) using the Center for Genomic Epidemiology (CGE) server (http://www.genomicepidemiology.org/services/), and the identification of antimicrobial resistance genes and acquired virulence factors by alignment against the Comprehensive Antibiotic Resistance Database (CARD) (https://card.mcmaster.ca) [29] and the Virulence Factors of Pathogenic Bacteria (VFDB) (http://www.mgc.ac.cn/VFs/main.htm) [30], respectively.

### Conjugation assays and S1-pulsed-field gel electrophoresis (S1-PFGE)

Conjugation assays were performed as described previously [31,32] using the ceftiofur-resistant subpopulations (EP70A, EP91A, EP174A, and EP184A) as donor strains and either rifampicin-resistant *E. coli* C600 or their corresponding susceptible subpopulations (EP70B, EP91B, EP174B, and EP184B) as recipient strains. Donor and recipient strains were mixed at a 1:1 ratio and allowed to mate for 6 h at 37°C. The mating mixtures were then serially diluted in 0.9% saline and plated onto selective MacConkey agar.

For conjugation assays using rifampicin-resistant *E. coli* C600 as the recipient, transconjugants were selected on MacConkey agar supplemented with ceftiofur (8 μg/mL) and rifampicin (200 μg/mL), followed by PCR detection of *bla*_CTX-M_. When the corresponding susceptible subpopulations were used as recipients, different selective and molecular screening strategies were applied according to the resistance profiles of each donor-recipient pair. For assays involving EP70B or EP91B as recipients, ceftiofur-containing MacConkey agar was used for initial screening, followed by PCR detection of *bla*_CTX-M_ and the corresponding plasmid replicon type. For assays involving EP174B as the recipient, MacConkey agar containing ceftiofur plus florfenicol was used. For assays involving EP184B as the recipient, MacConkey agar containing ceftiofur plus doxycycline was used. Candidate colonies from these assays were further screened by PCR detection of *bla*_CTX-M_ and the recipient-associated *qnrS1* marker to support the recipient background of candidate transconjugants. For each conjugation experiment, all candidate colonies from selective plates within the countable range of 30–200 colonies per plate were subjected to PCR screening for the relevant markers. Plasmid transfer in confirmed transconjugants was further verified by S1-PFGE.

Additionally, the minimum inhibitory concentrations (MICs) of various antimicrobial agents, including amoxicillin, ceftiofur, amikacin, doxycycline, florfenicol, gentamicin, colistin, and enrofloxacin, were determined for the PCHR subpopulations and transconjugants using the broth microdilution method in accordance with CLSI guidelines [22]. All antimicrobial agents were provided by Henan Muxiang Veterinary Pharmaceutical Co., Ltd. (Zhengzhou, China).

### Whole genome sequencing (WGS) analysis

Representative strains EP91A, EP91B and TEP91A were subjected to WGS using both the Illumina Nova-seq6000 platform (Illumina, Inc., San Diego, CA, USA) and the Oxford Nanopore MinION platform (Oxford Nanopore Technologies Ltd., Oxford, UK). The resulting short and long reads were hybrid-assembled using Unicycler v0.5.0 [33]. The assembled genomes were functionally annotated with the RAST v2.0 server (http://rast.nmpdr.org) [34]. Plasmid replicon genotypes were identified using PlasmidFinder 2.1 (https://cge.food.dtu.dk/services/PlasmidFinder/) and insertion sequences were detected via the ISfinder server (https://isfinder.biotoul.fr/). Genome comparisons and plasmid maps were visualized using Easyfig 2.2.3 and BRIG, respectively.

### Population dynamics of PCHR strains with and without ceftiofur pressure

Preserved purified cultures of each PCHR strain were diluted 1: 100 in LB broth and cultured until the OD_600_ reached 0.5. The culture was then subcultured at a 1: 100 ratio into one of the following conditions for continuous passage: (i) ceftiofur-pressure group, LB broth containing 8 μg/mL ceftiofur for 15 days; (ii) antibiotic-free group, drug-free LB broth for 30 days. Subculturing was performed every 24 h at the same dilution ratio.

After each passage, bacterial suspensions were serially diluted and plated onto both ceftiofur-containing (8 μg/mL ceftiofur) and antibiotic-free LB agar plates (Beijing Aoboxing Bio-Tech Co., Ltd., China). Following overnight incubation, colonies were counted. The proportion of resistant subpopulations was estimated from colony counts on ceftiofur-containing and antibiotic-free plates using the following formula: resistant subpopulation ratio = (number of colonies on ceftiofur-containing plates × corresponding dilution factor) / (number of colonies on antibiotic-free plates × corresponding dilution factor). These measurements were used to estimate changes in resistant-subpopulation frequency during serial passage.

Ceftiofur MICs of the passaged bacterial populations were determined by the broth microdilution method to monitor changes in ceftiofur susceptibility during serial passage. To assess whether resistance plasmid transfer occurred during serial passage, colonies grown on ceftiofur-containing plates were selected and examined using the same verification strategy as that used in the conjugation assays, including PCR detection of *bla*_CTX-M_, the corresponding plasmid replicon type, and recipient-associated markers when necessary.

### Growth curve analysis

Bacterial growth curves were determined by measuring the optical density at 600 nm (OD_600_). All strains, including the reference strain *E. coli* ATCC 25,922, were cultured in LB broth at 37°C with shaking. The OD_600_ was monitored at 1 h intervals for 14 h using a Multiskan SkyHigh microplate reader (Thermo Fisher Scientific, Waltham, MA, USA). To quantitatively compare growth profiles, growth parameters were calculated, including the area under the curve (AUC), maximum growth rate (μmax), and maximum OD_600_. AUC was calculated using the trapezoidal rule, μmax was calculated as the maximum slope between two consecutive measured time points, and maximum OD_600_ was defined as the highest OD_600_ value observed during the assay.

### In vitro competition experiments

Four pairs of resistant and susceptible subpopulations (EP91A/EP91B, EP70A/EP70B, EP174A/EP174B, and EP184A/EP184B) were mixed at a 1:1 ratio and co-cultured in LB broth. The cultures were serially passaged every 24 h at a 1:1000 dilution into fresh LB medium for a total of 6 days. At each 24 h interval, 100 μL of culture was collected, serially diluted 10-fold, and 100 μL aliquots were plated onto both ceftiofur-containing and antibiotic-free MacConkey agar plates. After 16 h of static incubation, colony-forming units (CFU) on each plate type were enumerated. The competitive index was calculated as the CFU of the susceptible subpopulation divided by the CFU of the resistant subpopulation [26], and plotted against cultivation time. Thus, a decreasing competitive index indicated an increasing relative abundance of the resistant subpopulation. The experiment was performed with three independent biological replicates.

### Plasmid stability assay

The transconjugants (TEP70A, TEP91A, TEP174A, TEP184A, TEP174F, and TEP184F) obtained from the conjugation assay were inoculated individually into LB broth and serially passaged every 24 h into fresh LB medium for 14 days under antibiotic-free conditions. Every 48 h, diluted bacterial cultures were spread onto both ceftiofur-containing and antibiotic-free LB agar plates. After incubation, colonies were counted on both plate types. Plasmid retention was estimated as the ratio of colonies grown on ceftiofur-containing plates to colonies grown on antibiotic-free plates according to previously described methods [35]. The experiment was performed with three independent biological replicates.

### Transcriptomic analysis

Total RNA was extracted from the resistant subpopulation EP91A and its susceptible counterpart EP91B using the RNAprep Pure Cell/Bacteria Kit (TIANGEN Biotech Co., Ltd., Beijing, China). RNA sequencing was performed on an Illumina NovaSeq 6000 platform (BGI Genomics Co., Ltd., Shenzhen, China). The resulting reads were aligned to the *E. coli* K-12 MG1655 reference genome (RefSeq accession no. CP025268). Mapping statistics for each sample are provided in Table S1. Differentially expressed genes (DEGs) were identified based on the fragments per kilobase of transcript per million mapped reads (FPKM) values, with a significance threshold of adjusted *p* < 0.05 and |log_2_(fold change)| >1. Based on the RNA-seq results, representative DEGs from enriched pathways were selected for primer design and further validation by RT-qPCR. Total RNA was extracted from independently prepared EP91A and EP91B cultures and reverse-transcribed into cDNA. RT-qPCR was performed using gene-specific primers, and relative expression levels were normalized to 16S rRNA and calculated using the 2^−ΔΔ^Ct method. The primers used for RT-qPCR are listed in Table S2.

### Data analyses

Statistical analyses were performed using GraphPad Prism v10.1.2 (GraphPad Software, Boston, MA, USA). Unless otherwise stated, data are presented as the mean ± standard deviation (SD), and *p* < 0.05 was considered statistically significant. Experiments were performed with at least three independent biological replicates unless otherwise indicated. For comparisons between two groups, two-tailed unpaired Welch’s t-tests were used when appropriate. For comparisons involving multiple groups, two-way ANOVA followed by Sidak’s multiple-comparisons test was used where applicable. Exact *p* values are reported where possible.

## Results

### Identification and validation of PCHR in swine-derived E. coli

**Figure 1.**
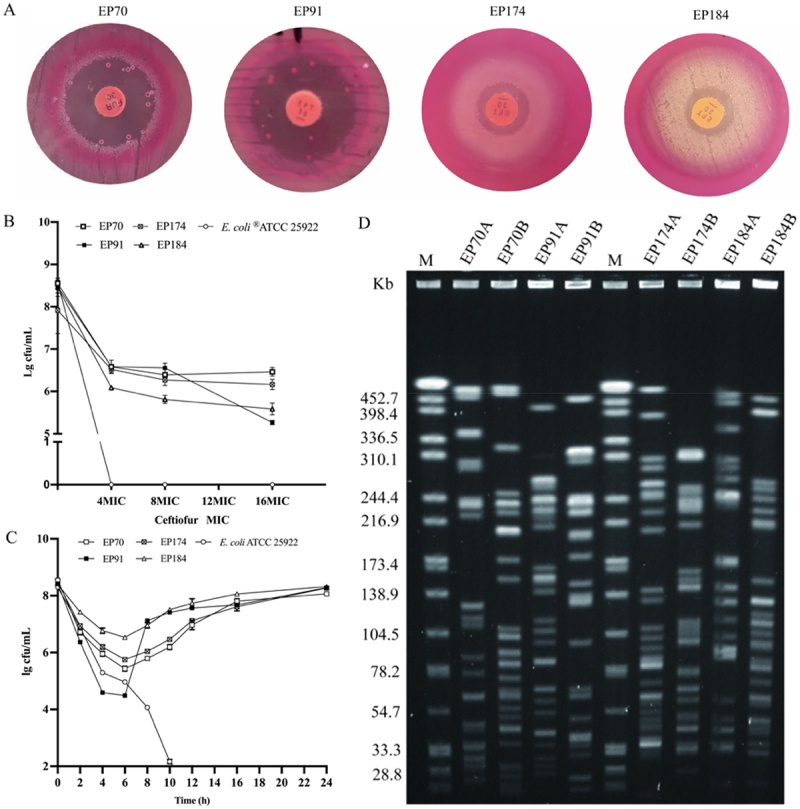
Identification and characterization of polyclonal ceftiofur heteroresistance (PCHR) in 453 swine-derived *E. coli* isolates. (A) heteroresistance phenotypes detected by disk diffusion assay. (B) population analysis profile (PAP) assays for isolates EP70, EP91, EP174, and EP184. (C) *in vitro* time-kill curves of ceftiofur against the PCHR isolates. (D) genetic relatedness assessed by pulsed-field gel electrophoresis (PFGE) of *XbaI*-digested genomic DNA. *E. coli* ATCC 25,922 served as the quality control strain. The ceftiofur-resistant subpopulations (designated EP70A, EP91A, EP174A, and EP184A; MIC = 512 μg/mL) and their susceptible counterparts (EP70B, EP91B, EP174B, and EP184B; MIC = 0.25 μg/mL) were isolated from the original PCHR isolates EP70, EP91, EP174, and EP184 (MIC = 0.25 μg/mL), respectively. M represents the molecular weight marker, *salmonella* serotype *braenderup* H9812.

Ceftiofur susceptibility testing showed that 74.8% (339/453) of the *E. coli* isolates were classified as susceptible, 18.9% (86/453) as resistant, and 6.3% (28/453) as intermediate according to CLSI criteria (Table 1). Notably, four isolates (EP70, EP91, EP174, and EP184) exhibited scattered colonies or a “halo” within the inhibition zones (Figure 1(A)), suggestive of a potential heteroresistant phenotype. Despite being categorized as susceptible (MIC = 0.25 μg/mL), these four isolates were selected for further investigation due to this atypical growth pattern.

**Table 1.** Drug sensitivity results detected by disk diffusion method.

Antimicrobial Agent	Resistant	Intermediate	Susceptible
Ceftiofur (30 μg)	≤17 mm	18–20 mm	≥21 mm
Number of strains	86	28	339
Proportion	18.9 %	6.3 %	74.8 %

PAP assays showed that all four isolates (EP70, EP91, EP174, and EP184), but not the reference strain *E. coli* ATCC 25,922, were able to grow on MHA plates containing ceftiofur at concentrations ≥ 4 × MIC (1 μg/mL). The frequency of resistant subpopulations ranged from 3.0 × 10^−3^ to 1.9 × 10^−2^ (Figure 1(B)). Time – kill assays further confirmed survival and persistence of these isolates under ceftiofur exposure (Figure 1(C)), supporting a heteroresistant phenotype. Accordingly, these four porcine-derived *E. coli* isolates were classified as CHR.

Within each isolate, the ceftiofur-resistant and -susceptible subpopulations were designated as EP70A/EP70B, EP91A/EP91B, EP174A/EP174B, and EP184A/EP184B, respectively. PFGE analysis showed distinct banding patterns between resistant and susceptible subpopulations within each isolate (Figure 1(D)), indicating genetic heterogeneity. These findings support that the isolates EP70, EP91, EP174, and EP184 exhibit PCHR.

### Whole-genome sequencing reveals genetic diversity and virulence gene profiles of PCHR strains

WGS analysis showed that resistant and susceptible subpopulations within each isolate belonged to distinct sequence types (STs) and serotypes (Table 2), consistent with a polyclonal origin. Specifically, the resistant subpopulations EP70A, EP91A, EP174A, and EP184A were assigned to O101: H10-ST617, O15: H18-ST69, O153: H25-ST3489, and O134: H21-ST345, respectively, indicating that they originated from genetically distinct lineages.

**Table 2.** Analysis of sequencing results for the four polyclonal ceftiofur heteroresistance (PCHR) strains.

Strains	Resistance genes	Virulence genes	MLST	Phylogenetic group	Serotypes	Inc types
EP70A	*bla*_CTX-M-55_, *bla*_TEM-1_, *mcr-1*, *floR*, *aac(3)-IId*, *aadA2b*, *catA2*, *sul3*, *dfrA12*, *tet*(A), *bleO*	*AslA*, *anr*, *csgA, fdeC*, *gad*, *hlyE, nlpI*, *terC*, *iss*, *traT*, *yehABCD*	617	A	O101: H10	IncFII, IncI2, IncX1
EP70B	*mcr-1*, *tet*(A), *bleO*	*csgA*, *fdeC, fimH*, *gad*, *hlyE*, *hra*, *lpfA*, *nlpI*, *terC*, *yehABCD*	898	A	O88: H48	IncFIB, IncI2
EP91A	*bla*_CTX-M-55_, *bla*_TEM-1_, *floR*, *aph(6)-Id*, *aph(3’’)-Ib*, *qnrS1*, *sul2*, *dfrA14*, *tet*(A)	*Air*, *AslA*, *Anr*, *cvaC*, *chuA*, *eilA*, *fimH*, *fyuA*, *gad*, *papA*, *iss*, *lpfA*, *ompT*, *sitA*, *terC*, *tia*, *hlyEF*, *iroN*, *iucC*, *iutA*, *mchF*, *traT*, *tsh*	69	D	O15: H18	IncFIB
EP91B	*bla*_TEM-176_, *aph(6)-Id*, *aph(3’’)-Ib*, *qnrS1*, *sul2*, *dfrA14*, *tet*(A)	*fimH*, *gad*, *terC*	746	A	O112: H18	IncFIB, IncX1,p0111
EP174A	*bla*_CTX-M-55_, *bla*_TEM-1_, *mcr-1*, *aac(3)-IId*, *aph(6)-Id*, *aph(3’”)-Ib*, *aph(3”)-Ia*, *rmtB*, *sul2*, *tet*(A), *bleO*	*AslA*, *csgA*, *fdeC*, *fimH*, *gad*, *hlyE*, *nlpI*, *terC*, *traT*, *yehABCD*	3489	A	O153: H25	IncFII, IncN
EP174B	*bla*_TEM-1_, *mcr-1*, *floR*, *aadA1*, *aac(3)-IId*, *aph(6)-Id*, *aph(3’”)-Ib*, *aph(3”)-Ia*, *aadA2b*, *rmtB*, *qnrS1*, *cmlA1*, *sul1, sul2, sul3*, *dfrA12*, *tet*(A), *bleO*	*AslA*, *csgA*, *fdeC*, *fimH*, *gad*, *hlyE*, *iss*, *nlpI*, *ompT*, *terC*, *traT*, *yehABCD*	3856	A	O100: H30	IncHI2, IncI1
EP184A	*bla*_CTX-M-14_, *bla*_TEM-1_, *mcr-1*, *aac(3)-IId*, *aadA2*, *aph(3’)-Ia*, *mph(A)*, *dfrA12*, *bleO*	*Air*, *cib*, *cma*, *csgA*, *cvaC*, *fdeC*, *fimH*, *gad*, *hlyE*, *iroN*, *iss*, *iucC*, *iutA*, *hlyEF*, *lpfA*, *nlpI*, *ompT*, *sitA*, *terC*, *traT*, *yehABCD*	345	B1	O134: H21	IncHIB, IncI1
EP184B	*bla*_TEM-1_, *mcr-1*, *floR*, *aac(3)-IId*, *aph(6)-Id*, *aph(3’”)-Ib*, *aadA1*, *aph(3”)-Ia*, *aadA2b*, *rmtB*, *qnrS1*, *lnu(F)*, *cmlA1*, *sul1, sul2, sul3*, *dfrA12*, *tet*(A), *bleO*	*anr*, *csgA*, *fimH*, *gad*, *hlyE*, *nlpI*, *sitA*, *terC*, *traT*, *yehABCD*	1421	A	O9: H4	IncHIB, IncI1

MLST, multilocus sequence typing; Inc, incompatibility.

Phylogenetic analysis further showed that the eight subpopulations were distributed across different phylogroups. EP91A belonged to phylogroup D and EP184A belonged to phylogroup B1, whereas the remaining subpopulations were assigned to phylogroup A. These results indicate phylogroup-level heterogeneity among the analyzed subpopulations, particularly in the EP91 and EP184 pairs.

Resistance gene profiling revealed differences among subpopulations. Except for EP70B, which carried only three resistance genes, the remaining subpopulations harbored 7–17 resistance-associated genes. Notably, all resistant subpopulations carried *bla*_CTX-M_, whereas this gene was absent in their susceptible counterparts, consistent with their ceftiofur resistance phenotypes. Interestingly, the susceptible subpopulations EP174B and EP184B carried a higher number of resistance genes (≥16) than their corresponding resistant subpopulations EP174A and EP184A (≤11). The biological basis of this difference was not further investigated in this study.

Virulence gene analysis showed that all subpopulations, except EP91B, carried multiple virulence-associated genes. EP91A and EP184A carried APEC-associated virulence genes, including *iutA, hlyF, iss*, *iroN*, and *ompT*, representing predicted virulence-associated genomic features [36,37]; however, functional pathogenicity was not assessed in this study. Collectively, these results highlight substantial heterogeneity in resistance and virulence-associated gene profiles among PCHR subpopulations.

### Comparative genomics identifies a ColV virulence plasmid and distinct resistance gene architectures in EP91 subpopulations

The resistant subpopulation EP91A (O15: H18, phylogroup D) contained a 4,979,631 bp chromosome and a single 180,964 bp plasmid (pEP91A-1), which was characterized as a ColV-like plasmid based on previously reported features [36]. In contrast, the susceptible subpopulation EP91B possessed a 4,561,780 bp chromosome and four plasmids (designated pEP91B-1 to pEP91B-4) ranging from 46 to 109 kb (Table 3).

**Table 3.** Genetic characteristics of plasmids in strains EP91A and EP91B.

Strains		GenBank	Resistance genes	Virulence genes	Inc types	Size (bp)
EP91A	chromosome	CP149815	*bla*_CTX-M-55_, *floR*, *aph(6)-Id*, *aph(3’’)-Ib*, *sul2*, *tet*(A)	*Air*, *AslA*, *chuA*, *eilA*, *fimH*, *fyuA*, *gad*, *iss*, *lpfA*, *ompT*, *papA_F19*, *sitA*, *terC*, *tia*, *hlyE*		4,979,631
	pEP91A-1	CP149816	*bla*_TEM-1_, *aph(6)-Id*, *aph(3’’)-Ib*, *qnrS1*, *sul2*, *tet*(A), *dfrA14*	*Anr*, *cvaC*, *etsC*, *hlyF*, *iroN*, *iss*, *iucC*, *iutA*, *mchF*, *ompT*, *sitA*, *traT*, *tsh*	IncFIB	180,964
EP91B	chromosome	CP149810	–	*fimH*, *gad*, *terC*	–	4,561,780
	pEP91B-1	CP149811	–	–	IncFIB	108,916
	pEP91B-2	CP149812	–	–	–	89,738
	pEP91B-3	CP149813	–	–	p0111	81,158
	pEP91B-4	CP149814	*bla*_TEM-176_, *floR*, *aph(3’)-Ia*, *qnrS1*, *tet*(A), *dfrA14*	–	IncX1-	46,569

Inc, incompatibility.

Resistance gene profiles were analyzed to describe genomic differences between the two subpopulations. Both EP91A and EP91B harbored multiple resistance genes, consistent with a multidrug-resistant (MDR) background. In EP91A, six resistance genes (*bla*_CTX-M-55_, *floR*, *aph(6)-Id*, *aph(3”‘)-Ib*, *sul2* and *tet*(A)) were located on the chromosome, whereas the ColV plasmid pEP91A-1 carried seven additional resistance genes (*bla*_TEM-1_, *aph(6)-Id*, *aph(3’”)-Ib*, *qnrS1*, *sul2*, *tet*(A), *dfrA14*). In contrast, resistance genes in EP91B were exclusively plasmid-borne, with pEP91B-4 harboring *bla*_TEM-176_, *floR*, *aph(3’)-Ia*, *qnrS1*, *tet(*A), and *dfrA14*.

**Figure 2.**
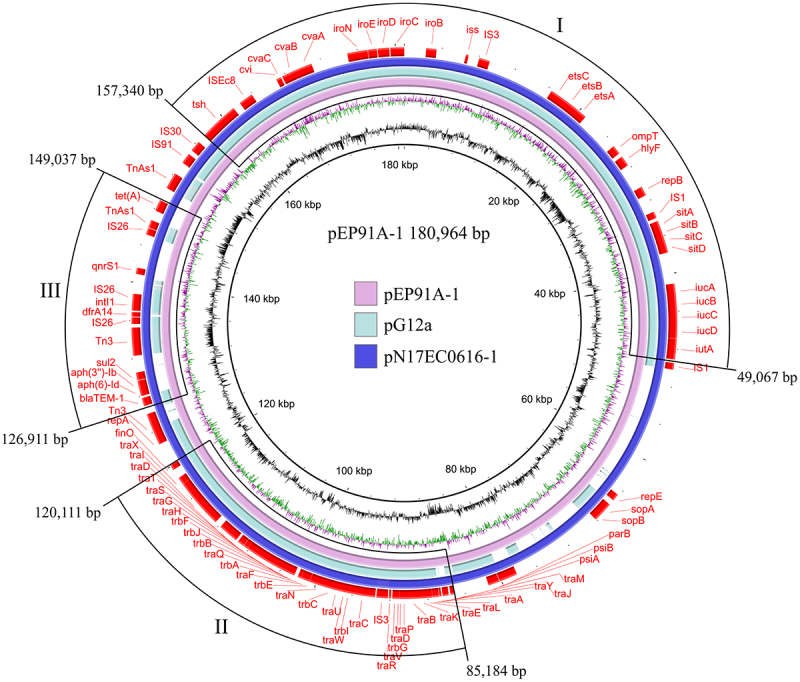
Circular comparison of plasmid pEp91A-1 with plasmids pG12a and pN17EC0616-1. The outer ring represents the annotated sequence of pEp91A-1, with the red box highlighting the region shown in detail. Gaps indicate regions with low or no sequence similarity compared with the reference plasmid pEp91A-1. Region I represents the ColV-associated virulence region, region II represents the conjugative transfer region, and region III represents the multidrug resistance region (MRR).

Further characterization showed that pEP91A-1 was an IncFIB plasmid with high sequence identity (99.96%) to pG12a, a ColV-positive IncFIB/IncFIC plasmid previously reported in avian-derived *E. coli* [36]. Comparative analysis indicated that pEP91A-1 contained an expanded multidrug resistance region (MRR), including additional resistance genes (*aph(6)-Id*, *aph(3’’)-Ib*, *qnrS1*, and *sul2*), as well as a distinct genomic context of *tet*(A), compared with pG12a (Figure 2). In addition, pEP91A-1 shared high sequence identity (98.7%) with pN17EC0616-1, an IncF plasmid identified in retail chicken isolates in the United States [37], indicating genetic relatedness to plasmids circulating in animal-associated *E. coli* populations.

### Transferability and maintenance of bla_CTX-M_-carrying plasmids in E. coli

**Figure 3.**
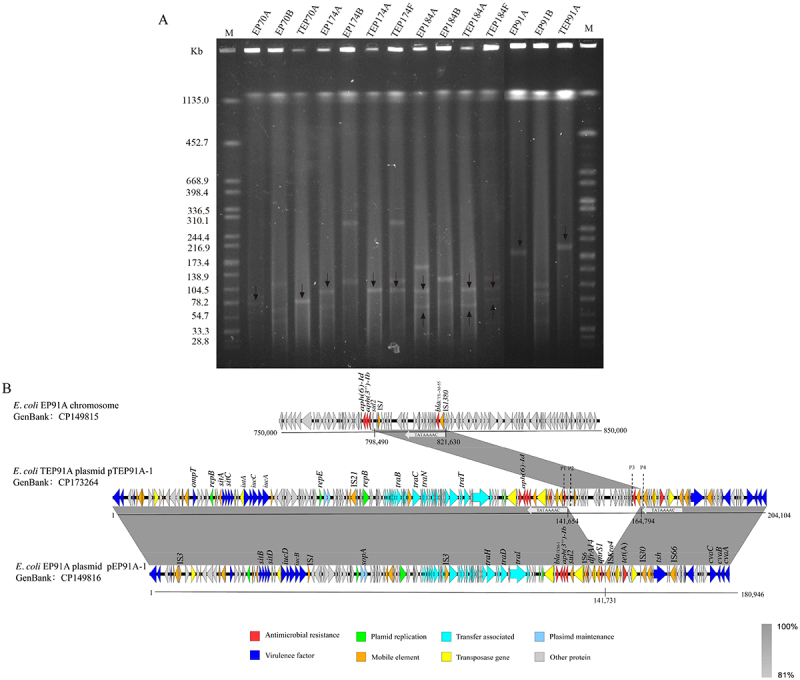
Plasmid-mediated dissemination of *bla*_CTX-M_. (A) S1-pulsed-field gel electrophoresis (S1-PFGE) analysis of genomic DNA from transconjugants and their corresponding donor and recipient strains. (B) linear comparison of the plasmid pEp91A-1 with the corresponding chromosomal region of EP91A and the transconjugant plasmid pTep91A-1. Primer pairs P1/P2 and P3/P4 indicate the primers used to amplify the left and right junction regions of the 23,140-bp insertion, respectively. The gray arrows indicate the position and orientation of the 8-bp homologous sequence/site (TATAAAAC). M, molecular weight marker (*salmonella* serotype *braenderup* H9812). The ceftiofur-resistant (EP70A, EP91A, EP174A, EP184A) and susceptible (EP70B, EP91B, EP174B, EP184B) subpopulations were isolated from the corresponding polyclonal ceftiofur heteroresistance (PCHR) isolates EP70, EP91, EP174, and EP184, respectively. Transconjugants TEP70A, TEP91A, TEP174A, and TEP184A were obtained by conjugating these resistant subpopulations with *E. coli* C600. Additionally, transconjugants TEP174F and TEP184F were specifically generated by conjugating donors EP174A and EP184A with their susceptible counterparts, EP174B and EP184B, respectively.

Conjugation assays showed that *bla*_CTX-M_-carrying plasmids from all four resistant subpopulations were transferable to *E. coli* C600, generating transconjugants TEP70A, TEP91A, TEP174A, and TEP184A, respectively, although with markedly different frequencies. EP70A, EP174A, and EP184A exhibited relatively high conjugation frequencies, whereas EP91A showed a significantly lower frequency of (1.23 ± 0.16) × 10^−7^. Candidate transconjugants were confirmed by PCR detection of *bla*_CTX-M_, and plasmid transfer was further verified by S1-PFGE (Table 4; Figure 3(A)).

**Table 4.** Conjugation frequency of resistant subpopulations.

Donors	Recipients	Transconjugants	Transfer rates
EP70A	*E. coli* C600	TEP70A	(1.58 ± 0.24) × 10^−2^
EP70A	EP70B	TEP70F	N
EP91A	*E. coli* C600	TEP91A	(1.23 ± 0.16) × 10^−7^
EP91A	EP91B	TEP91F	N
EP174A	*E. coli* C600	TEP174A	(3.02 ± 0.28) × 10^−2^
EP174A	EP174B	TEP174F	(2.27 ± 0.18) × 10^−2^
EP184A	*E. coli* C600	TEP184A	(1.49 ± 0.94) × 10^−3^
EP184A	EP184B	TEP184F	(7.81 ± 0.27) × 10^−3^

All experiments were performed with biological replicates and presented as the mean ± standard deviation (SD).

N indicates that the transconjugants were not successfully obtained.

When the corresponding susceptible subpopulations (EP70B, EP91B, EP174B, and EP184B) were used as recipients, transconjugants were obtained only for EP174A and EP184A, designated TEP174F and TEP184F, respectively. No verified transconjugants were detected for EP70A or EP91A under the tested conditions. The corresponding conjugation frequencies for EP174A and EP184A were comparable to those observed using *E. coli* C600 as the recipient (Table 4; Figure 3(A)).

Antimicrobial susceptibility testing further confirmed acquisition of *bla*_CTX-M_-carrying plasmids in all six transconjugants, as evidenced by markedly increased ceftiofur MICs (512 μg/mL) and acquisition of resistance to both ceftiofur and amoxicillin (Table S3).

To assess plasmid maintenance, stability assays were performed in all transconjugants. The *bla*_CTX-M_-carrying plasmids in TEP70A, TEP91A, TEP174A, TEP184A, TEP174F, and TEP184F were maintained at high levels during 14-day serial passage in antibiotic-free medium, with retention rates ranging from 90% to 100% (Figure S1A). These observations indicate that *bla*_CTX-M_-carrying plasmids can be stably maintained once established, which may contribute to their persistence in the absence of antibiotic selection.

### IS1380-associated chromosomal-plasmid recombination and potential contribution to bla_CTX-M_ mobilization

Comparative genomic analysis was performed to examine plasmid structural changes associated with the unusually low conjugation frequency observed in EP91A. The plasmid in the transconjugant TEP91A (pTEP91A-1) was approximately 20 kb larger than the donor plasmid pEP91A-1. Despite high overall sequence similarity, a distinct 23,140 bp insertion was identified at positions 141,654–164,794 in pTEP91A-1 (Figure 3(B)). This inserted fragment showed high sequence similarity to a chromosomal region of the donor strain EP91A and contained the *bla*_CTX-M_ gene together with its flanking sequences.

Further sequence analysis indicated that the insertion region was associated with an IS*1380* element present in the EP91A chromosome. To validate the predicted insertion junctions specific to pTEP91A-1, two primer pairs, P1/P2 and P3/P4, were designed to amplify the left and right junction regions of the 23,140-bp insertion, respectively (Figure 3(B) and Table S4). PCR amplification generated products of the expected sizes, 1,832 bp for P1/P2 and 2,920 bp for P3/P4, in TEP91A, whereas no corresponding products were detected in EP91A or *E. coli* C600 (Figure S2). Sanger sequencing of the PCR products confirmed that both amplicons matched the predicted junction regions of pTEP91A-1, supporting the presence and boundary structure of the 23,140-bp insertion.

Together, these results support the presence of a chromosomal fragment carrying *bla*_CTX-M_ within pTEP91A-1 and suggest that IS*1380* may be involved in recombination at an 8-bp homologous site (TATAAAAC) within plasmid pEP91A-1. The resulting plasmid exhibited increased size and structural complexity compared with the donor plasmid, which may be associated with the reduced conjugation frequency observed in EP91A, although alternative explanations cannot be excluded. These findings are consistent with a possible chromosome-to-plasmid mobilization event involving IS*1380*-associated recombination and may represent a potential route for *bla*_CTX-M_ dissemination.

### Divergent population-level processes underlying resistance expansion under ceftiofur pressure

**Figure 4.**
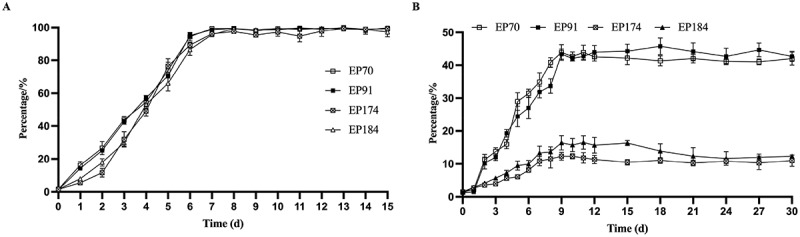
Proportion of *bla*_CTX-M_-harboring subpopulations during serial passage with or without ceftiofur selection. PCHR isolates were cultured with (A) or without (B) ceftiofur selection. Data are presented as mean ± standard deviation (SD) from three independent biological replicates.

Under sustained ceftiofur pressure (8 μg/mL), all four PCHR strains exhibited marked expansion of resistant subpopulations, with resistant fractions exceeding 94.5% by day 7 (Figure 4(A)). This consistent enrichment suggests a strong selective effect of ceftiofur, although the underlying population dynamics differed among strains.

**Figure 5.**
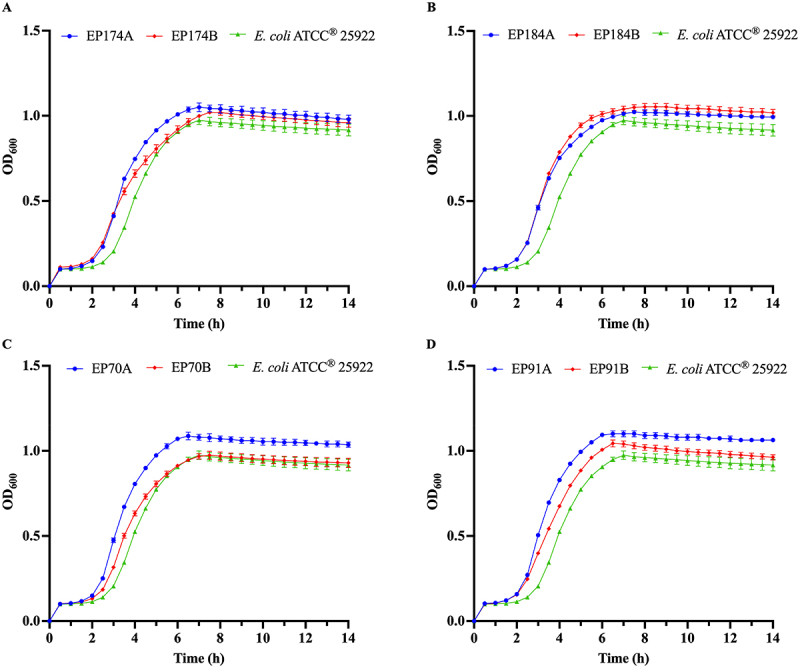
Growth curves of resistant and susceptible subpopulations from polyclonal ceftiofur heteroresistance (PCHR) isolates. Growth curves of resistant and susceptible subpopulations from EP174 (A), EP184 (B), EP70 (C), and EP91 (D), with *E. coli* ATCC 25,922 included as the reference strain. Data represent mean ± standard deviation (SD) of three biological replicates. Quantitative growth parameters and statistical comparisons based on two-tailed unpaired Welch’s t-tests are summarized in table S5.

In EP174 and EP184, PCR-based verification of colonies obtained from ceftiofur-containing plates on day 8 showed that previously susceptible subpopulations had acquired resistance plasmids, indicating that resistance expansion was associated with plasmid transfer events. Growth-parameter analysis showed that these two strain pairs did not exhibit a consistent resistant-subpopulation growth advantage comparable to that observed in EP70 and EP91 (Figure 5(A,B) and Table S5). For EP174A and EP174B, AUC values were 10.99 ± 0.21 and 10.54 ± 0.05, respectively (*p* = 0.054), and maximum OD_600_ values were 1.050 ± 0.026 and 1.023 ± 0.012, respectively (*p* = 0.217), whereas EP174A showed a higher μmax than EP174B (0.437 ± 0.013 vs 0.337 ± 0.017, *p* = 0.00174). For EP184A and EP184B, no significant differences were observed in AUC (10.91 ± 0.12 vs 11.29 ± 0.21, *p* = 0.066), μmax (0.413 ± 0.013 vs 0.417 ± 0.008, *p* = 0.733), or maximum OD_600_ (1.023 ± 0.012 vs 1.053 ± 0.021, *p* = 0.113). These results are consistent with a contribution of horizontal transfer to resistance expansion in EP174 and EP184.

In contrast, EP70 and EP91 showed no evidence of plasmid acquisition in susceptible subpopulations. Growth-parameter analysis showed that EP70A and EP91A had significantly higher AUC, μmax, and maximum OD_600_ values than their susceptible counterparts EP70B and EP91B, respectively (Figure 5(C,D) and Table S5). Specifically, EP70A showed higher AUC (11.52 ± 0.19 vs 10.06 ± 0.18, *p* = 0.00063), μmax (0.449 ± 0.010 vs 0.370 ± 0.033, *p* = 0.043), and maximum OD_600_ (1.087 ± 0.023 vs 0.975 ± 0.020, *p* = 0.0035) than EP70B. Similarly, EP91A showed higher AUC (11.80 ± 0.15 vs 10.74 ± 0.16, *p* = 0.00113), μmax (0.468 ± 0.011 vs 0.305 ± 0.005, *p* = 0.000287), and maximum OD_600_ (1.100 ± 0.017 vs 1.043 ± 0.021, *p* = 0.0235) than EP91B. In line with these growth-related differences, resistant subpopulations rapidly increased in proportion and displaced their susceptible counterparts within 5–6 days in competition assays (Figure S1B), indicating differential expansion of preexisting resistant subpopulations under antibiotic pressure.

Together, these findings suggest that ceftiofur exposure consistently enriches resistant subpopulations in PCHR strains, while resistance expansion may occur through different population-level processes, including plasmid transfer and differential expansion of preexisting resistant subpopulations.

### Population dynamics of PCHR strains under antibiotic-free conditions

Under antibiotic-free passage, ceftiofur MICs of the passaged bacterial populations increased rapidly in all four PCHR strains, reaching 512 μg/mL within 6 days, corresponding to a 2048-fold increase relative to the initial MIC. This change was consistent with the expansion of resistant subpopulations during serial passage.

The resistant subpopulations exhibited two distinct expansion patterns (Figure 4(B)). EP70 and EP91 reached higher proportions rapidly and stabilized at approximately 43% of the population, whereas EP174 and EP184 increased more gradually and remained at lower levels of approximately 12–16%. Consistent with the growth-parameter analysis described above, the higher maintenance levels observed for EP70 and EP91 were associated with more pronounced growth-related differences between resistant and susceptible subpopulations, whereas EP174 and EP184 showed less consistent growth-related differences (Figure 5(A-D) and Table S5).

PCR-based verification of colonies obtained from ceftiofur-containing plates on day 10 showed no evidence of *bla*_CTX-M_ plasmid transfer to susceptible subpopulations under antibiotic-free conditions. Collectively, these results indicate that, in antibiotic-free environments, the population dynamics of PCHR strains varied among isolates and were associated with clone-associated growth profiles and competitive interactions between subpopulations.

### Clone-associated transcriptional differences between EP91A and EP91B

**Figure 6.**
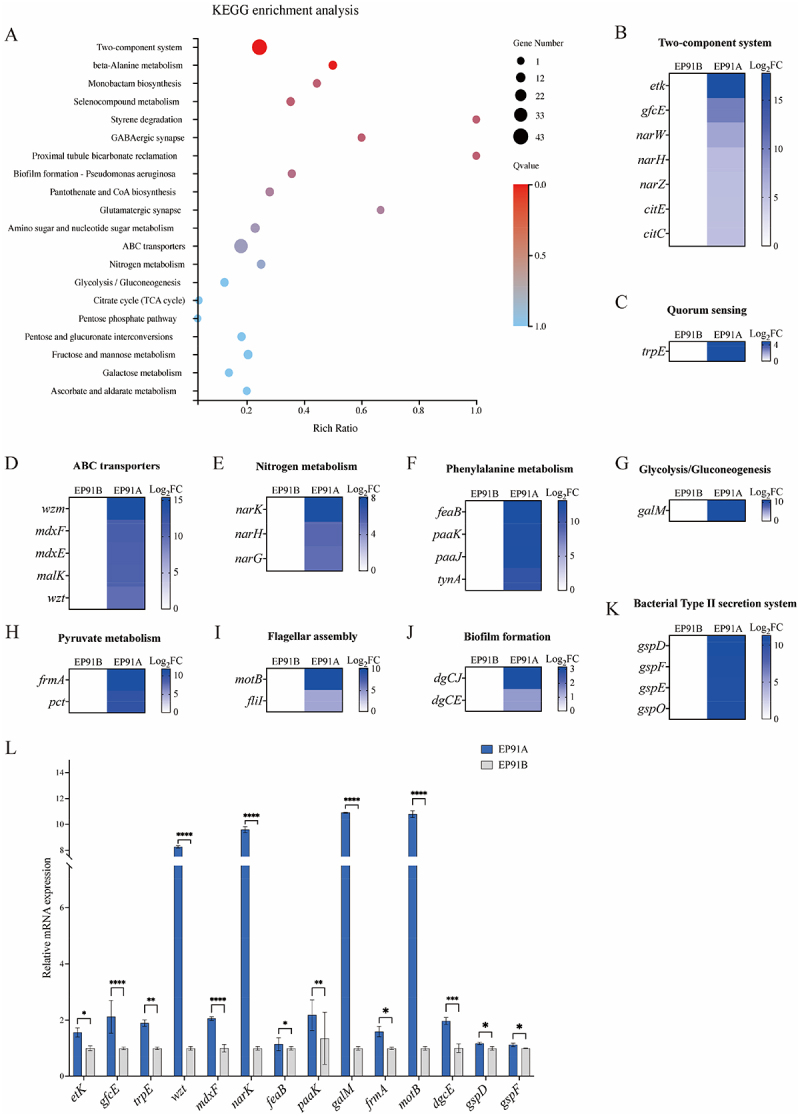
Transcriptomic profiling of clone-associated expression differences between EP91A and EP91B. (A) Kyoto encyclopedia of genes and genomes (KEGG) pathway enrichment analysis of differentially expressed genes (DEGs). Expression changes of DEGs in pathways related to the two-component system (B), quorum sensing (C), ABC transporters (D), nitrogen metabolism (E), phenylalanine metabolism (F), glycolysis/gluconeogenesis (G), pyruvate metabolism (H), flagellar assembly (I), biofilm formation (J), and the bacterial type II secretion system (K), RNA-seq analysis was performed with three biological replicates per group. (L), RT-qPCR validation of selected representative DEGs from enriched pathways. Data in panel L are presented as mean ± standard deviation (SD) from three biological replicates. Statistical comparisons for panel L were performed using two-way ANOVA followed by Sidak’s multiple-comparisons test. *p* < 0.05 was considered statistically significant. Asterisks indicate significant differences (*p < 0.05; **p < 0.01; ***p < 0.001; ****p < 0.0001).

To explore clone-associated transcriptional differences between the resistant subpopulation EP91A and its susceptible counterpart EP91B, transcriptomic analysis was performed. A total of 736 differentially expressed genes (DEGs) were identified, including 286 upregulated and 450 downregulated genes in EP91A (Figure S3). KEGG pathway enrichment analysis showed that these DEGs were distributed across multiple functional categories, including environmental sensing and regulatory systems, nutrient transport, metabolism, and cellular processes (Figure 6(A)).

Specifically, EP91A showed upregulation of genes associated with two-component systems (Figure 6(B)), quorum sensing (Figure 6(C)), and ABC transporters (Figure 6(D)). Genes involved in several metabolic pathways, including nitrogen metabolism (Figure 6(E)), phenylalanine metabolism (Figure 6(F)), glycolysis/gluconeogenesis (Figure 6(G)), and pyruvate metabolism (Figure 6(H)), were also enriched among the upregulated DEGs. In addition, pathways related to cellular behavior and interaction, including flagellar assembly (Figure 6(I)), biofilm formation (Figure 6(J)), and the bacterial type II secretion system (Figure 6(K)), were enriched. RT-qPCR analysis of selected representative DEGs from these enriched pathways showed expression trends consistent with the RNA-seq results, with higher expression levels in EP91A than in EP91B (Figure 6(L)).

Together, these results indicate broad differences in gene expression profiles between EP91A and EP91B. Given that EP91A and EP91B represent genetically distinct clones rather than isogenic resistant and susceptible variants, these transcriptomic differences, although not direct evidence for the molecular mechanism underlying PCHR, nevertheless reflect clone-associated transcriptional differences and provide hypothesis-generating observations for future functional studies.

## Discussion

The extensive use of antibiotics in veterinary medicine has accelerated the emergence of bacterial resistance, with heteroresistance increasingly recognized as a clinically relevant yet frequently overlooked phenomenon. Heteroresistance can allow bacterial populations to survive antimicrobial exposure and may facilitate the transition toward stable resistance, thereby complicating therapeutic outcomes [21]. Within this context, our study provides evidence that PCHR can occur in swine-derived *E. coli* isolates, highlighting a previously underappreciated layer of population complexity in veterinary pathogens.

A key feature of the PCHR phenotype identified in this study is the coexistence of genetically distinct resistant and susceptible subpopulations within the same initial isolate culture. This polyclonal structure differs from the more commonly described MHR driven by within-clone variation [8,21], and instead suggests the coexistence of multiple lineages with distinct genetic backgrounds. Such population heterogeneity has been reported to compromise the accuracy of routine antimicrobial susceptibility testing, as minor resistant subpopulations can remain undetected while retaining the capacity to expand under selective pressure [38].

Consistent with current epidemiological trends, *bla*_CTX-M_ genes were detected in all resistant subpopulations, with *bla*_CTX-M-55_ predominating. This observation aligns with reports describing the increasing prevalence and dissemination of *bla*_CTX-M-55_ in China and other regions [39], supporting its important contribution to ceftiofur resistance in the resistant subpopulations analyzed here. The co-occurrence of APEC-associated virulence genes (*iutA*, *hlyF*, *iss*, *iroN* and *ompT*) in certain resistant subpopulations is consistent with previous findings in avian pathogenic *E. coli* (APEC) [40], suggesting a potential pathogenic capacity of the resistant subpopulations, although further studies will be valuable to clarify their functional roles and epidemiological significance.

Previous studies have attributed heteroresistance to mechanisms such as point mutations, insertions or small deletions, biofilm-associated protection, *β*-lactamase gene copy number variation and transient genetic alterations [41,42]. In the present study, comparative genomic analysis, together with junction PCR and Sanger sequencing, supported the presence of a *bla*_CTX-M_-containing chromosomal fragment inserted into pTEP91A-1. This structure is consistent with a possible IS*1380*-associated recombination event, suggesting that insertion sequences may contribute to the movement of resistance gene fragments from the chromosome to plasmids. The role of IS elements in resistance gene dynamics has been increasingly recognized [43,44]. In this context, our results are consistent with a putative chromosome-to-plasmid mobilization event involving IS-associated recombination, although alternative explanations cannot be excluded and further experimental validation is required to confirm the underlying recombination process.

Our results further suggest that resistance expansion in PCHR populations may be context-dependent. Under ceftiofur exposure, resistance expansion showed different patterns among the four isolates. In EP174 and EP184, previously susceptible subpopulations were found to carry *bla*_CTX-M_-bearing plasmids after exposure, suggesting a role for plasmid-mediated horizontal transfer. In contrast, in EP70 and EP91, no plasmid acquisition was observed in susceptible subpopulations, while resistant subpopulations increased in relative abundance, consistent with differential expansion of preexisting resistant clones under antibiotic pressure. This pattern is consistent with previous studies showing that both horizontal gene transfer and clonal selection can contribute to resistance emergence under antibiotic pressure [45].

In the absence of antibiotics, resistant subpopulations remained detectable but were maintained at different levels among strains, reflecting strain-specific population dynamics. These differences suggest that the maintenance of resistant subpopulations in PCHR isolates may be influenced not only by resistance determinants, but also by clone-associated growth differences and competitive interactions among coexisting subpopulations [46]. Given that the resistant and susceptible subpopulations represent genetically distinct clones, these patterns should be interpreted as clone-associated population dynamics rather than as a single, uniform resistance-related fitness effect.

At the transcriptomic level, RNA-seq analysis revealed broad differences in gene expression profiles related to environmental sensing, metabolism, and cellular processes in EP91A compared with EP91B. RT-qPCR analysis of selected representative DEGs supported the expression trends observed in the RNA-seq data. However, because EP91A and EP91B represent genetically distinct subpopulations rather than isogenic resistant and susceptible variants, these transcriptional differences should not be interpreted as direct mechanistic determinants of heteroresistance. Instead, they reflect clone-associated transcriptional variation between two coexisting clones. Similar global transcriptional remodeling has been reported in bacteria under antibiotic exposure and other environmental stresses [47]. Therefore, the observed multi-pathway differences should be considered exploratory and hypothesis-generating, and further functional studies will be required to determine whether any of these pathways contribute to the phenotypic differences observed in this study.

Collectively, our findings suggest that PCHR in these selected swine-derived *E. coli* isolates is a multifaceted phenomenon associated with genetic heterogeneity, transferable *bla*_CTX-M_-carrying plasmids, possible mobile-element-associated gene movement, and strain-dependent population dynamics. The coexistence of plasmid-mediated transfer and clone-associated differential expansion, together with a putative chromosome-to-plasmid mobilization event, highlights multiple layers of complexity in ceftiofur resistance dynamics. These findings emphasize the need for improved detection of heteroresistance and for surveillance strategies that consider both horizontal gene transfer and the maintenance of resistant subpopulations in veterinary and public health contexts.

## Supplementary Material

Supplementary_material (1)clean.docx

## Data Availability

The genome sequencing data supporting the findings of this study are publicly available in NCBI database (https://www.ncbi.nlm.nih.gov). The BioSample accession numbers for strains EP70A, EP70B, EP174A, EP174B, EP184A, and EP184B range from SAMN47582960 to SAMN47582965. The genome sequences of strains EP91A, EP91B, and TEP91A have been deposited under GenBank accession numbers CP149815-CP149816, CP149810-CP149814, CP173263-CP173264, respectively. The raw RNA-sequencing data are available in the NCBI Sequence Read Archive (SRA) under BioProject ID PRJNA1210467 (https://www.ncbi.nlm.nih.gov/search/all/?term = PRJNA1210467). Detailed BioProject, BioSample, Assembly, WGS/GenBank, SRA Experiment, and SRA Run accession information for the genome assemblies and RNA-seq datasets is provided in Tables S6 and S7. The source data supporting the figures and tables presented in this study are openly available in Figshare at https://doi.org/10.6084/m9.figshare.31860925 [48].
