# Supplementary material for: Dual population-level processes contribute to polyclonal ceftiofur heteroresistance in swine-derived Escherichia coli
Source: Virulence. 2026 Jul 30;17(1):2711528. doi: 10.1080/21505594.2026.2711528 (PMC13432863; doi:10.1080/21505594.2026.2711528)
Supplement: Supplementary_material (1)clean.docx [file KVIR_A_2711528_SM0381.docx]

**Table S1 Mapping statistics of RNA-seq reads against the *E. coli* K-12 MG1655 reference genome.**

| Sample | Total clean reads (M) | Total genome mapping (%) | Uniquely genome mapping (%) |
| --- | --- | --- | --- |
| EP91A | 11.28 | 96.37 | 95.69 |
| EP91B | 11.25 | 96.55 | 91.87 |

**Table S2 Primer sequences used for RT-qPCR validation of representative differentially expressed genes (DEGs).**

| Gene | Primer sequences (5′-3′) | Length (bp) | Reference |
| --- | --- | --- | --- |
| *etK* | F: ACTCAGCCGCAGCCAGTGAA | 147 | this study |
|  | R: GCCGTGCTCTTCCAGTTGTTCC |  |  |
| *gfcE* | F: CGAAGGACGCATCGGCAATGAT | 103 | this study |
|  | R: ATCACACGGTTCCAACGAGCCA |  |  |
| *trpE* | F: ACGTCTCACTGCTCGCCTGAAC | 132 | this study |
|  | R: CGCACTACGCCACCGAACTCTT |  |  |
| *wzt* | F: GCGTGGGCAGCAACTTCCTTT | 168 | this study |
|  | R: CGATCAAGAGTCCGTGCGTACC |  |  |
| *mdxF* | F: CGTCCATCGTTGCGGCTTCAT | 142 | this study |
|  | R: GCATGGCTCACCGATCCGTTCT |  |  |
| *narK* | F: TTCTCGGCGTCAATGGCGTACC | 165 | this study |
|  | R: AGACAGGGAGCTGGTCGGCAAT |  |  |
| *feaB* | F: GCAACATCGTCAGTGGCGTGGT | 131 | this study |
|  | R: TGGCGTAGTGGCGGGAATTGTG |  |  |
| *paak* | F: CCGACACGCCTTGCCTTCTGAA | 116 | this study |
|  | R: GTTGCCAGGTATGCCACCAGCT |  |  |
| *galm* | F: CCAGCATTCAGCGTCGCATTCC | 150 | this study |
|  | R: TCCGCATGATGGTGCCAGGAT |  |  |
| *frmA* | F: ATCTGTTCCGCACCGAGCAACC | 123 | this study |
|  | R: AACGGCATGGCAGGCAGCAA |  |  |
| *motB* | F: TGACACGACGAGAGCGATTAGG | 112 | this study |
|  | R: GCGACACACCAACAATGGGTT |  |  |
| *dgcE* | F: ACTCCCACTCCCAGGTGCCAAT | 171 | this study |
|  | R: CGTTGCTTGCCGTCTCACTGGT |  |  |
| *gspD* | F: TGCTGCTTCTCTGTGCCGATGA | 153 | this study |
|  | R: CCTGCTCCGCCAGATGATGGAT |  |  |
| *gspF* | F: TGCGTGTACTCAGCGGCAGTTG | 111 | this study |
|  | R: TGCTCACTACGGTGGCGATTGG |  |  |
| *16s RNA* | F: TGTCTGGGAAACTGCCTGATGG | 159 | this study |
|  | R: TCGCCTAGGTGAGCCGTTAC |  |  |

**Table S3 The MIC results for transconjugants and their parental strains by antimicrobial susceptibility test (μg/mL).**

| Strains | Ceftiofur | Amoxicillin | Colistin | Florfenicol | Gentamicin | Enrofloxacin | Amikacin | Doxycycline |
| --- | --- | --- | --- | --- | --- | --- | --- | --- |
| *E. coli* C600 | 0.25 | 8 | 0.01 | 2 | 0.125 | 0.125 | 0.25 | 0.5 |
| EP70A | 512 | >512 | 2 | 128 | 64 | 256 | 1 | 32 |
| TEP70A | 512 | >512 | 0.25 | 2 | 0.125 | 0.25 | 0.25 | 0.25 |
| EP70B | 0.25 | 16 | 2 | 2 | 0.5 | 0.01 | 1 | 16 |
| EP91A | 512 | >512 | 2 | 64 | 0.5 | 0.125 | 1 | 16 |
| TEP91A | 512 | >512 | 0.5 | 2 | 0.25 | 0.25 | 1 | 2 |
| EP91B | 0.25 | >512 | 2 | 128 | 0.5 | 0.25 | 1 | 8 |
| EP174A | 512 | >512 | 2 | 16 | >512 | 8 | >512 | 16 |
| TEP174A | 512 | >512 | 0.25 | 4 | >512 | 0.125 | 0.25 | 2 |
| EP174B | 0.25 | >512 | 2 | 128 | >512 | 1 | >512 | 16 |
| TEP174F | 512 | >512 | 2 | 128 | >512 | 8 | >512 | 16 |
| EP184A | 512 | >512 | 2 | 4 | 64 | 32 | 1 | 2 |
| TEP184A | 512 | >512 | 0.5 | 4 | 0.25 | 0.25 | 0.25 | 1 |
| EP184B | 0.25 | >512 | 2 | 128 | >512 | 0.5 | >512 | 32 |
| TEP184F | 512 | >512 | 2 | 128 | >512 | 0.5 | >512 | 32 |

**Table S4 Primer sequences used for junction PCR validation of the predicted insertion boundaries in pTEP91A-1.**

| Primer pair | Primer sequences (5′-3′) | PCR product (bp) | Reference |
| --- | --- | --- | --- |
| P1 | F: TCACCTTCACCGCTTGCACATT | 1832 | this study |
| P2 | R: TTGCTCCGCACTACCGTTCTG |  |  |
| P3 | F: AGCCGCCGACGCTAATACATC | 2920 | this study |
| P4 | R: GCAGGTCTGGCATCAGCAAGA |  |  |

P1-P2 and P3-P4 were used to amplify the left and right junction regions of the 23,140-bp insertion in pTEP91A-1, respectively.

**Table S5 Quantitative growth parameters of resistant and susceptible subpopulations.**

| Subpopulation | AUC | μmax | Maximum OD_600_ |
| --- | --- | --- | --- |
| EP70A | 11.52 ± 0.19 | 0.449 ± 0.010 | 1.087 ± 0.023 |
| EP70B | 10.06 ± 0.18 | 0.370 ± 0.033 | 0.975 ± 0.020 |
| *P* value | 0.00063 | 0.043 | 0.0035 |
| EP91A | 11.80 ± 0.15 | 0.468 ± 0.011 | 1.100 ± 0.017 |
| EP91B | 10.74 ± 0.16 | 0.305 ± 0.005 | 1.043 ± 0.021 |
| *P* value | 0.00113 | 0.000287 | 0.0235 |
| EP174A | 10.99 ± 0.21 | 0.437 ± 0.013 | 1.050 ± 0.026 |
| EP174B | 10.54 ± 0.05 | 0.337 ± 0.017 | 1.023 ± 0.012 |
| *P* value | 0.054 | 0.00174 | 0.217 |
| EP184A | 10.91 ± 0.12 | 0.413 ± 0.013 | 1.023 ± 0.012 |
| EP184B | 11.29 ± 0.21 | 0.417 ± 0.008 | 1.053 ± 0.021 |
| *P* value | 0.066 | 0.733 | 0.113 |

Data are presented as mean ± standard deviation (SD) from three independent biological replicates. the area under the curve (AUC) was calculated using the trapezoidal method. The maximum growth rate (μmax) was estimated as the maximum ΔOD_600_/Δt between two adjacent time points. *P* values were calculated using two-tailed unpaired Welch’s t-tests comparing resistant and susceptible subpopulations within each strain pair. A value of *P* < 0.05 was considered statistically significant.

**Table S6 NCBI accession information for genome assemblies used in this study.**

| Strain | BioProject | BioSample | Assembly | WGS accession | GenBank accession |
| --- | --- | --- | --- | --- | --- |
| EP70A | PRJNA1242196 | [SAMN47582960](https://www.ncbi.nlm.nih.gov/biosample/SAMN47582960) | [GCA_049202865.1](https://www.ncbi.nlm.nih.gov/datasets/genome/GCA_049202865.1/) | [JBMIQQ000000000](https://www.ncbi.nlm.nih.gov/nuccore/JBMIQQ000000000) | NA |
| EP70B |  | [SAMN47582961](https://www.ncbi.nlm.nih.gov/biosample/SAMN47582961) | [GCA_049197845.1](https://www.ncbi.nlm.nih.gov/datasets/genome/GCA_049197845.1/) | [JBMIQP000000000](https://www.ncbi.nlm.nih.gov/nuccore/JBMIQP000000000) | NA |
| EP174A |  | [SAMN47582962](https://www.ncbi.nlm.nih.gov/biosample/SAMN47582962) | [GCA_049199505.1](https://www.ncbi.nlm.nih.gov/datasets/genome/GCA_049199505.1/) | [JBMIQO000000000](https://www.ncbi.nlm.nih.gov/nuccore/JBMIQO000000000) | NA |
| EP174B |  | SAMN47582963 | [GCA_049202935.1](https://www.ncbi.nlm.nih.gov/datasets/genome/GCA_049202935.1/) | [JBMIQN000000000](https://www.ncbi.nlm.nih.gov/nuccore/JBMIQN000000000) | NA |
| EP184A |  | [SAMN47582964](https://www.ncbi.nlm.nih.gov/biosample/SAMN47582964) | [GCA_049202855.1](https://www.ncbi.nlm.nih.gov/datasets/genome/GCA_049202855.1/) | [JBMIQM000000000](https://www.ncbi.nlm.nih.gov/nuccore/JBMIQM000000000) | NA |
| EP184B |  | [SAMN47582965](https://www.ncbi.nlm.nih.gov/biosample/SAMN47582965) | [GCA_049197985.1](https://www.ncbi.nlm.nih.gov/datasets/genome/GCA_049197985.1/) | [JBMIQL000000000](https://www.ncbi.nlm.nih.gov/nuccore/JBMIQL000000000) | NA |
| EP91A | PRJNA1087166 | [SAMN40435517](https://www.ncbi.nlm.nih.gov/biosample/SAMN40435517) | [GCA_037939535.1](https://www.ncbi.nlm.nih.gov/datasets/genome/GCA_037939535.1/) | NA | CP149815-CP149816 |
| EP91B | PRJNA1087160 | [SAMN40435416](https://www.ncbi.nlm.nih.gov/biosample/SAMN40435416) | [GCA_037938425.1](https://www.ncbi.nlm.nih.gov/datasets/genome/GCA_037938425.1/) | NA | CP149810-CP149814 |
| TEP91A | PRJNA1178886 | [SAMN44485242](https://www.ncbi.nlm.nih.gov/biosample/SAMN44485242) | [GCA_044905065.1](https://www.ncbi.nlm.nih.gov/datasets/genome/GCA_044905065.1/) | NA | CP173263-CP173264 |

WGS, whole-genome shotgun; NA, not applicable.

**Table S7 NCBI accession information for RNA-seq datasets used in this study.**

| Strain | BioProject | BioSample | SRA Experiment | SRA Run |
| --- | --- | --- | --- | --- |
| EP91A | PRJNA1210467 | SAMN40435517 | SRX27345219 | SRR31990279 |
| EP91B |  | SAMN40435416 | SRX27345220 | SRR31990278 |


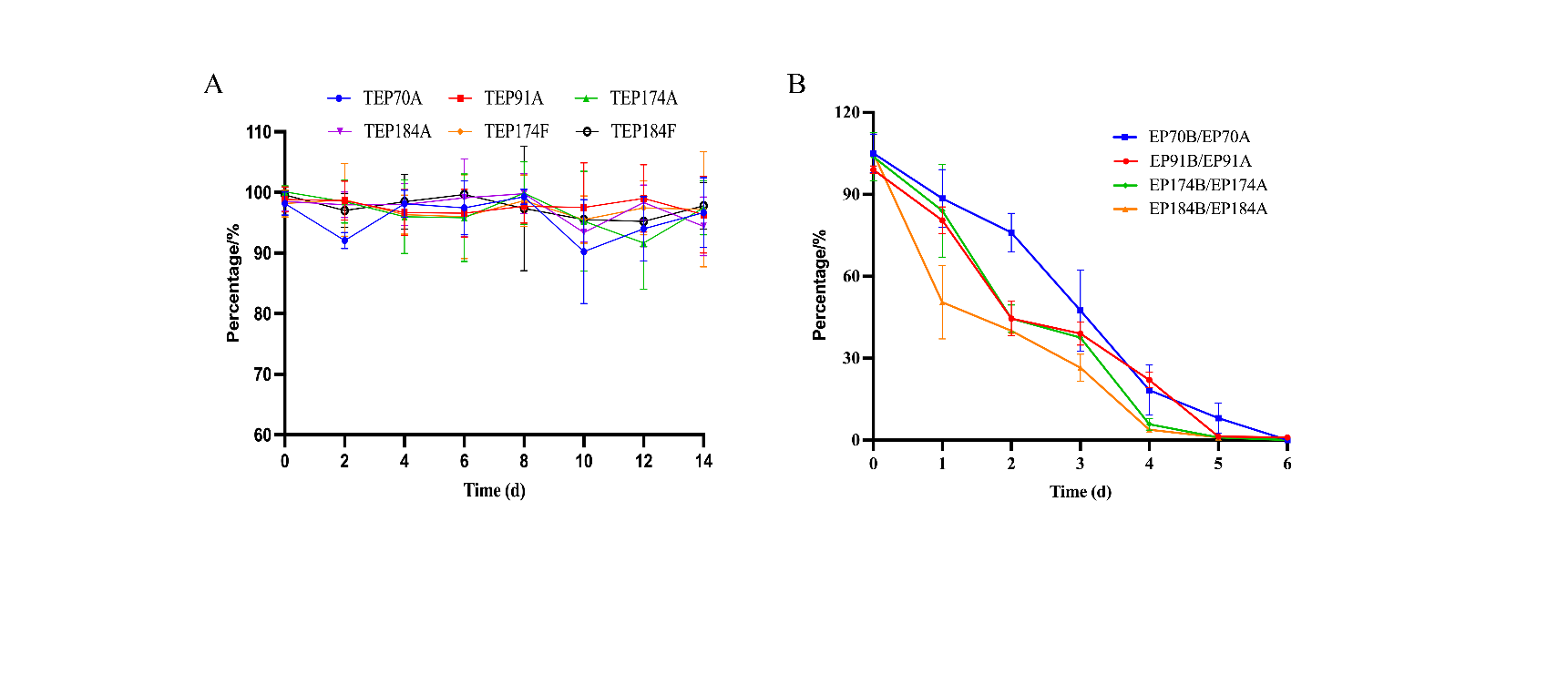


Figure S1 Plasmid stability and competitive dynamics of *bla*_CTX-M_-harboring strains. (A) Stability of *bla*_CTX-M_-harboring plasmids in six transconjugants during 14-day serial passage in antibiotic-free medium. (B) Competitive index between susceptible and resistant subpopulations from a normalized 1:1 starting ratio. The ceftiofur-resistant (EP70A, EP91A, EP174A, EP184A) and susceptible (EP70B, EP91B, EP174B, EP184B) subpopulations were isolated from the corresponding polyclonal ceftiofur heteroresistance strains. Transconjugants TEP70A, TEP91A, TEP174A, and TEP184A were obtained by conjugating resistant subpopulations with *E. coli* C600, while TEP174F and TEP184F were generated by mating EP174A and EP184A with their susceptible counterparts EP174B and EP184B, respectively. Data represent mean ± standard deviation (SD) from three independent experiments.


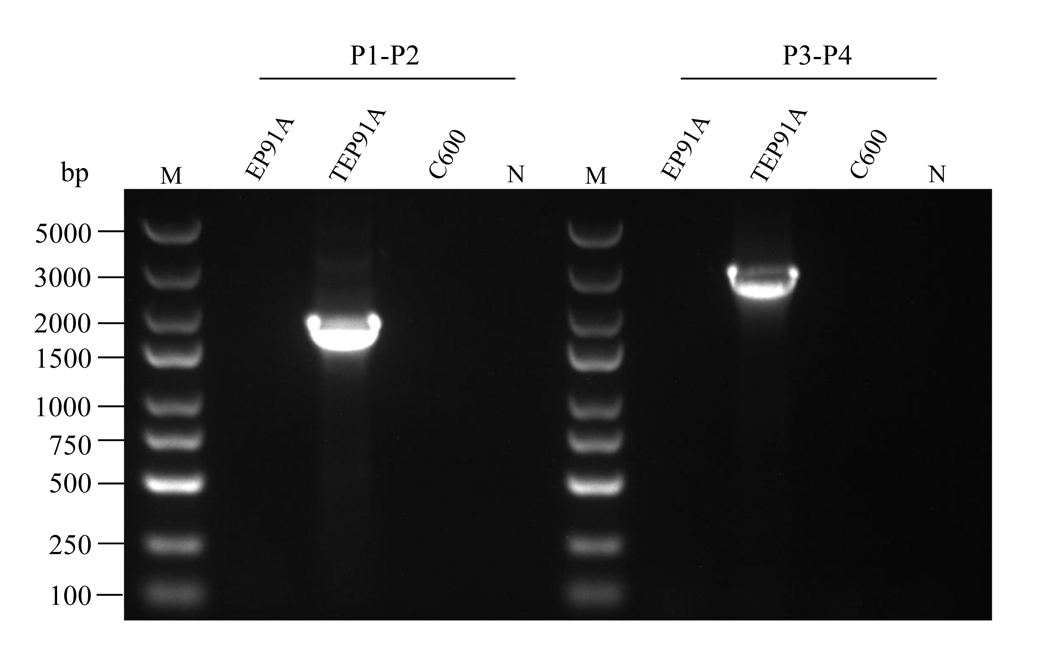


Figure S2 Junction PCR validation of the predicted insertion boundaries in pTEP91A-1. Primer pairs P1/P2 and P3/P4 were designed to amplify the left and right junction regions of the 23,140-bp insertion, respectively. Amplicons of the expected sizes, 1,832 bp for P1/P2 and 2,920 bp for P3/P4, were detected in TEP91A but not in EP91A, *E. coli* C600, or the negative control. Sanger sequencing of the PCR products confirmed that the amplicons matched the corresponding junction regions of pTEP91A-1. M, DNA marker; N, negative control.


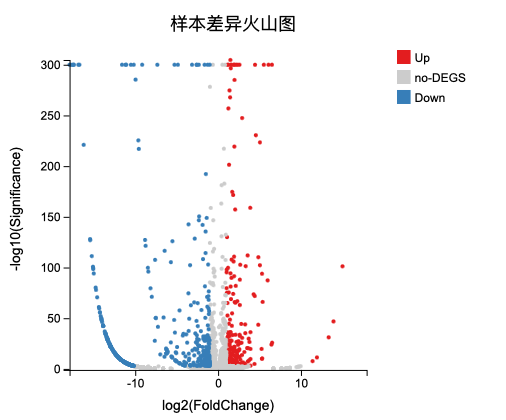


Figure S3 Statistics of DEGs between the resistant subpopulation EP91A and the susceptible subpopulation EP91B.
